# Pembrolizumab-induced Stevens-Johnson syndrome/Toxic Epidermal Necrolysis in a Vietnamese patient with nonsmall-cell lung cancer

**DOI:** 10.5415/apallergy.0000000000000131

**Published:** 2023-12-18

**Authors:** Yen T.H. Pham, Mai T. Vu, Anh Q. Nguyen, Phat N. Trinh, Mai H. Tran, Hieu C. Chu, Nguyet T.M. Nguyen, Chi H.V. Vu, Dinh V. Nguyen

**Affiliations:** 1 Center of Allergy and Clinical Immunology, Vinmec Times City, Vinmec Healthcare System, Hanoi, Vietnam; 2 College of Health Sciences, VinUniversity, Hanoi, Vietnam; 3 Center of Biomedical Informatics, Vingroup Big Data Institute, Hanoi, Vietnam; 4 Center of allergy and Clinical Immunology, Bach Mai Hospital, Hanoi, Vietnam; 5 Department of Allergy and Immunology, inmec-VinUni Institute of Immunology, Vinmec Healthcare System, Hanoi, Vietnam; 6 Cornea department, National Ophthalmology Hospital, Hanoi, Vietnam; 7 Department of Medicine, Penn State University, PA, USA

**Keywords:** Adverse drug reaction, case report, corticosteroids, immune checkpoint inhibitors, pembrolizumab, Stevens-Johnson syndrome

## Abstract

Chemoimmunotherapy is an effective therapy for an individual with nonsmall-cell lung cancer (NSCLC) without anaplastic lymphoma kinase or epidermal growth factor receptor mutations. However, it can also be related to adverse cutaneous reactions such as Stevens-Johnson syndrome (SJS) and toxic epidermal necrolysis (TEN) with high morbidities and mortality rates. We present a case of a 65-year-old male with NSCLC who underwent first-line chemotherapy with paclitaxel, carboplatin, and pembrolizumab, which was later followed by a second cycle of the same therapies. The patient developed a fever and rash 12 days after the second cycle. Pembrolizumab was strongly suspected as the culprit medication because cutaneous reactions to this drug have been frequently reported and threw other medications used as less likely candidates. This is the first case reported in Vietnam of SJS/TEN related to pembrolizumab and contributes to our knowledge of severe skin reactions associated with immune checkpoint inhibitors.

## 1. Introduction

Stevens-Johnson syndrome (SJS) and toxic epidermal necrolysis (TEN) are life-threatening, severe drug eruptions, characterized by blisters and generalized epidermolysis. A safety analysis of clinical trials and the food and drug administration (FDA) pharmacovigilance database documented 411 cases of SJS (n = 253) or TEN (n = 184) related to immune checkpoint inhibitors (ICIs) therapy, indicating that ICIs are associated with an increased risk of SJS/TEN. This increased risk also exists for the ICI pembrolizumab, which is a monoclonal against PD-1 (programmed cell death protein 1) [1]. A literature review reported 32 cases of SJS/TEN caused by ICI including 11 cases related to pembrolizumab [2]. In Vietnam, however, no case of SJS/TEN associated with pembrolizumab has been reported. This is also the first case of SJS/TEN in a nonsmall-cell lung cancer (NSCLC) patient receiving chemoimmunotherapy with pembrolizumab.

## 2. Case report/case presentation

**Figure 1.**
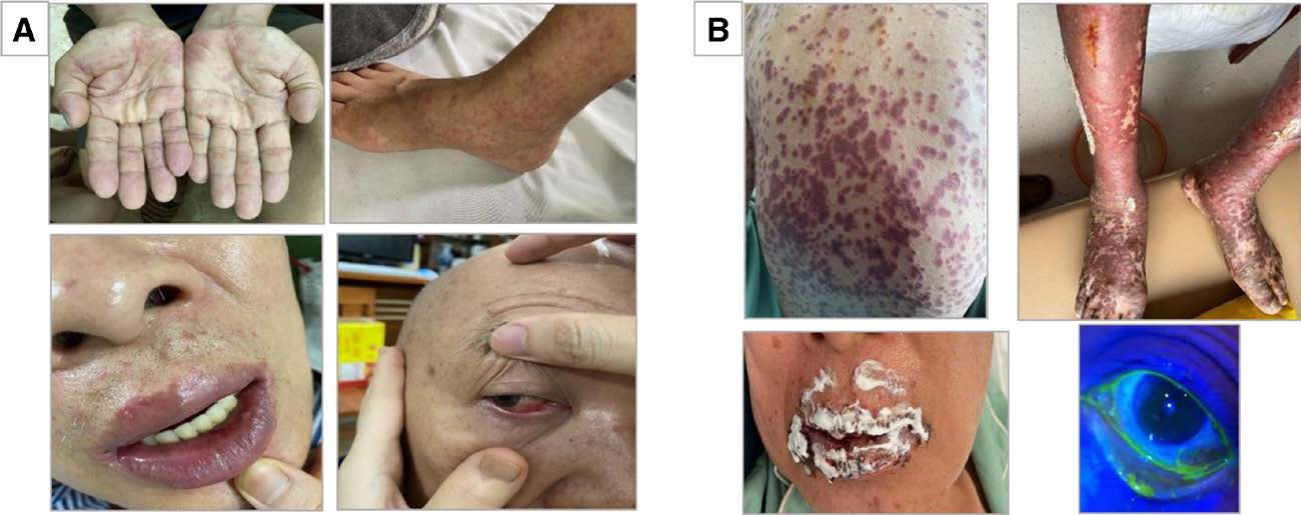
Serious disorder of the patient’s mucosa and skin. (A) Skin and mucosa lesions at the time of disease onset. (B) Progressive mucosal and skin damage.

A 65-year-old male with a history of NSCLC without anaplastic lymphoma kinase or epidermal growth factor receptor mutations received treatment with paclitaxel and carboplatin for the first chemotherapy cycle and transitioned to first-line chemoimmunotherapy with the addition of pembrolizumab 4 weeks later (Supplementary Table 1, http://links.lww.com/PA9/A19). After 12 days of treatment, he developed a few scattered atypical erythematous lesions all over the body including his palms. He then developed mucosal involvement with mouth ulceration, sore throat, and conjunctivitis, along with a fever of 38°C (Fig. 1A). The patient was admitted to our hospital with a diagnosis of SJS suspected as being due to pembrolizumab (Table 1) with a Score of Ten (SCORTEN) of 3 points (ages >40; heart rates, 120 beats/minute; cancer). The initial ocular examination showed conjunctival hyperemia, small conjunctival and eyelid epithelial loss without membrane, and no corneal epithelial defect. His blood tests showed normal liver and kidney functions and an increased inflammatory response (procalcitonin was 0.0928 [normal value <0.05]). His blood tests also showed that he had been infected with EBV, CMV, HSV1, and HSV2 but we did not see any reactivation of these viruses. He was treated immediately with intravenous immunoglobulin (IVIG) at a dose of 0.4 mg/kg/d for 3 days, intravenous methylprednisolone (2 mg/kg/d), and intravenous with paracetamol for pain relief, fluids, and optimal topical treatment of eyes and mouth. Despite treatment, his skin rash continued to spread and worsen (Fig. 1B). He developed new bullae scattered all over the body, a positive Nikolsky sign and genital ulcers; all of which fulfilled the criteria for a diagnosis of SJS/TEN. At that time, he had 4 points on SCORTEN including age, heart rate, cancer, and a body surface area (BSA) of skin detachment more than 10% but less than 30%. He was given 2 more days of IVIG treatment and methylprednisolone continued for a further 10 days, together with meticulous care of his damaged skin and mucous membranes of his eyes and genitalia. The following ocular examination showed improvement of all ocular surface involvement. Unfortunately, on the 8th day of his illness, he developed a skin infection with *Staphylococcus argenteus*, which was sensitive to several antibiotics and he was treated with cefazolin 1g every 8 hours for 7 days. Bacterial blood cultures were negative. One month from the onset of his SJS/TEN, his skin and ocular lesions had almost completely healed. He only had a healing genital erosion when discharged.

**Table 1.** ALDEN score for the patient’s medication used

Criterion	Value	Rule to apply	Medication							
			Palitaxel	Carboplatin	Pembrolizumab	Dexamethasone	Granisetron	Pregabalin	Etoricoxib	Esomeprazole
Delay from initial drug component intake to onset of reaction (index day)	Suggestive: +3	From 5 to 28 d			3					
	Compatible: +2	From 29 to 56 d	2	2		2	2	2	2	2
	Likely: +1	From 1 to 4 d								
	Unlikely: −1	>56 d								
	Excluded: −3	Drug started on or after the index day								
Drug present in the body on index day	Definite: 0	Drug continued up to index day or stopped at a time point <5× the elimination half-life before the index day			0					
	Doubtful: −1	Drug stopped at a time point prior to the index day by more than 5× the elimination half-life but liver or kidney function alterations or suspected drug interactions are present								
	Excluded: −3	Drug stopped at a time point prior to the index day by more than 5× the elimination half-life, without liver or kidney function alterations or suspected drug interactions	−3	−3		−3	−3	−3	−3	−3
Prechallenge/rechallenge	Positive specific for disease and drug: 4	SJS/TEN after use of same drug								
	Positive specific for disease or drug: 2	SJS/TEN after use of similar drug or other reaction with same drug								
	Positive unspecific: 1	Other reaction after use of similar drug								
	Not done/unknown: 0	No known previous exposure to this drug	0	0	0	0	0	0	0	0
	Negative: −2	Exposure to this drug without any reaction (before or after reaction)								
Dechallenge	Dechallenge	Drug stopped (or unknown)	0	0	0	0	0	0	0	
	Negative: −2	Drug continued without harm								−2
Type of drug (notoriety)	Strongly associated: 3	Drug of the “high-risk” list according to previous case–control studies								
	Associated: 2	Drug with definite but lower risk according to previous case–control studies								
	Suspected: 1	Several previous reports, ambiguous epidemiology	1		1				1	1
	Unknown: 0	All other drugs including newly released ones		0		0	0			
	Not suspected: −1	No evidence of association from previous epidemiology study with sufficient number of exposed controls						−1		
Other cause	Possible: −1	Rank all drugs from highest to lowest intermediate score	0	0	0	0	0	0	0	0
Final score: −12 to 10			0	−1	4	−1	−1	−2	0	−2

SJS/TEN, Stevens-Johnson syndrome/toxic epidermal necrolysis.

## 3. Discussion/Conclusions

The exact mechanism of SJS/TEN remains undetermined. The currently recognized theory is that it is a T-cell-mediated type IV delayed hypersensitivity reaction that causes keratinocyte apoptosis with epidermal necrosis and dermoepidermal separation [10]. The blockade PD-1 of pembrolizumab can contribute to an imbalance in the immune system, manifesting as an enhancement of the T-cell response and increasing the incidence of hypersensitivity [11].

Our patient was clinically diagnosed with SJS/TEN based on the lesions in the oral mucosa and ocular surface, eyelid, genital ulceration, and the occurrence of skin detachment of from 10% to 30% of BSA. Most patients with SJS/TEN are diagnosed clinically. Skin histology is only indicated in difficult cases.

Pembrolizumab, an ICI, is an immunotherapeutic agent targeting the PD-1 and is increasingly used in the treatment of NSCLC [12]. Therefore, the frequency of reports of drug-related serious skin reactions has also increased (Table 2). The first pembrolizumab-induced SJS case in NSCLC was reported in a 69-year-old Japanese male patient who was receiving first-line treatment with pembrolizumab for lung cancer, and 12 days after the first administration of pembrolizumab, the patient developed a fever and oral mucosa erosions [13]. Our case report is the first Vietnamese case of pembrolizumab-associated SJS/TEN in NSCLC. Our patient received treatment with paclitaxel and carboplatin for the first chemotherapy cycle and later transitioned to first-line chemoimmunotherapy with the addition of pembrolizumab 4 weeks later. Index day for the SJS/TEN was also 12 days from the treatment onset of pembrolizumab. The algorithm of drug causality for epidermal necrolysis (ALDEN) score [14, 15] was determined for all suspected drugs. ALDEN scores for pembrolizumab, paclitaxel, and carboplatin were, respectively, 4 points, −1 point, and −1 point (Table 1). These results indicate that pembrolizumab is the most probable cause of SJS/TEN in this case.

**Table 2.** Demographic, clinical features and treatment of pembrolizumab therapy-induced SJS/TEN in previously reported cases

Reference	Age	Sex	Oncologic history	Site affected	Index day	Diagnosis	Treatment	Outcome
Goldinger et al. [3]	77	M	Melanoma	Oral mucosa, trunk, genitals	-	SJS	Systemic steroids	-
Saw et al. [4]	50	F	Nasopharyngeal carcinoma	Conjunctiva Head/neck, trunk, arms	140 d (after the 5th cycle)	SJS	Prednisolone Cyclosporine	Stable
Saw et al. [4]	53	M	Renal cell carcinoma	Oral mucosa hands, foot, back	77 d (before 3rd cycle)	SJS	Cyclosporine	Resolution after 3 wk Switched to targeted therapy
Storandt and Seth [5]	55	F	Nonsmall-cell lung cancer	Most of body Oral mucosa Conjunctiva	4 mo	SJS/TEN	Intravenous methylprednisolone IVIG	Stable
Hwang et al. [6]	55	F	Melanoma	Oral mucosa hand foot	4 mo (9 cycles)	SJS	Prednisone 1 mg/kg/d	Stable
Haratake et al. [13]	69	M	Lung cancer	Oral mucosa; Conjunctiva Arms	12 d	SJS	Prednisolone	Stable after 30 d
Kumar et al. [7]	57	F	Metastatic lung adenocarcinoma	Oral mucosa Whole body	14 d	TEN	Methylprednisolone Plasmapheresis Infliximab	-
Robinson et al. [8]	55	F	Cervical squamous cell carcinoma	Oral mucosa; Conjunctiva Trunk, upper extremity, lower extremity	17 d	SJS/TEN	Methylprednisolone	Stable after 1 mo
Wu et al. [9]	68	F	Squamous cell carcinoma of the lung	Oral mucosa; conjunctiva Head, neck, chest, and back	20 d	SJS	Intravenous methylprednisolone (2 mg/kg/d) γ- globulin 20 g/d Sepprayi 25 mg	Stable after 3 mo

IVIG, intravenous immunoglobulin; SJS/TEN, Stevens-Johnson syndrome/toxic epidermal necrolysis.

In conclusion, chemoimmunotherapy introduced in the 2010s has revolutionized oncology therapy and the ICI pembrolizumab has an established benefit in the treatment of progressive lung cancer. Due to its increased use, the number of reports of pembrolizumab-induced SJS/TEN is increasing. SJS/TEN caused by pembrolizumab should be considered if a patient has new onset fever, skin rash with erythema multiforme, sore throat, and ocular involvement, as early recognition will lead to early treatment, which will reduce morbidity and mortality. Moreover, the combination of high-dose corticosteroid and IVIG has proven effective as a rescue therapy in the treatment pembrolizumab-induced severe SJS/TEN.

Although the number of reported cases are few, we found that our patient had many similar characteristics to reported clinical cases, especially in lung cancer patients, regarding clinical aspects and index day (Table 2). In a literature review that analyzed 32 cases of SJS/TEN caused by ICI, 11/32 cases were related to pembrolizumab. SJS/TEN usually occurred in the first or second cycle of treatment. The earliest onset occurred after 8 days, and the latest at 78 days after first administration [2]. A safety analysis of clinical trials and the FDA pharmacovigilance database included 20 randomized control trials (11,597 patients), which showed that ICIs were associated with an increased risk of SJS/TEN (odds ratio [OR] = 4.33; 95% confidence interval, 1.90–9.87). FDA adverse event reporting system pharmacovigilance data identified 411 cases of SJS (n = 253) or TEN (n = 184) related to ICI therapy [1]. These data suggest that ICI including pembrolizumab were significantly associated with an increased risk of SJS/TEN.

There is no standard treatment regimen for SJS/TEN. Multidisciplinary care and meticulous supportive care is advised for all SJS and/or TEN patients. Currently, combined corticosteroids and IVIG are considered the most effective therapy [16, 17]. Because our patient had severe, rapidly progressive skin lesions and a SCORTEN of 4 points, IVIG, intravenous methylprednisolone, and intensive supportive care were administered early at the time of the diagnosis of SJS/TEN. In the previously reported cases of pembrolizumab-associated SJS/TEN, most patients were prescribed corticosteroids with IVIG being added in severe cases (SJS/TEN).

A numbers of drug-induced severe cutaneous adverse reaction that bind to the T-cell receptor and the major histocompatibility complex class I have been reported. As highlighted in Vietnamese population, human leukocyte antigen (HLA), class I, B*15:02 and HLA-B*58:01 are strongly associated with SJS/TEN in treatment of aromatic, antiepileptic drugs (carbamazepine, oxcarbazepine) (OR, 33.78 [7.55–151.03], *P* < 0.0001) [18] and allopurinol (OR, 143.1 [32.3–634.5], *P* < 0.0001) [19], respectively. Interestingly, our patient’s HLA genotype contained risk alleles of ADRs with HLA-B*38:01, which was found related to cotrimoxazole-induced SJS/TEN and HLA-B*35:05 was associated with skin adverse drug reaction in Thai human immunodeficiency virus-infected patients on nevirapine treatment [15, 20] (Supplementary Table 2, http://links.lww.com/PA9/A19). However, there is no study that investigated the association between ICIs-induced SJS/TEN and HLA system, suggesting the need to have further study in order to uncover the cellular mechanism of pembrolizumab-induced SJS/TEN.

## Acknowledgments

We thank the patient and his family for their permission to report his case, including his deidentified clinical photographs. We thank Mr. Thanh H. Nguyen and Mr. Minh D. Vu at High-tech center for helping us in whole-exome sequencing analysis. We thank Vinmec Healthcare System Biobank for storing DNA samples. This work was partly supported by professors: Craig J. Timothy and Nunen A.V. Sheryl for writing-reviewing and editing.

## Conflict of interest

The authors have no financial conflicts of interest.

## Author contributions

Yen T.H. Pham: diagnosis and treatment, data analysis, conceptualization, writing-original draft. Nguyet T.M. Nguyen: discussion, writing-original draft. Mai T. Vu, Anh Q. Nguyen, Phat N. Trinh, Chi H.V. Vu: diagnosis, data analysis. Hieu C. Chu: diagnosis. Mai H. Tran: whole-exome sequencing analysis. Dinh V. Nguyen: conceptualization, diagnosis, writing-reviewing and editing, funding acquisition.

## Supplementary material

Supplementary Tables 1 and 2 can be found via http://links.lww.com/PA9/A19

Supplementary Tables 1 and 2

Click here to view

## Supplementary Material



## References

[1] Zhu J Chen G He Z Zheng Y Gao S Li J Ling Y Yu X Qiu K Wu J. Stevens-Johnson syndrome/toxic epidermal necrolysis in patients treated with immune checkpoint inhibitors: a safety analysis of clinical trials and FDA pharmacovigilance database. EClinicalMedicine. 2021;37:100951. 34386743 10.1016/j.eclinm.2021.100951 PMC8343267

[2] Hu L Nomura S Sato Y Takagi K Ishii T Honma Y Watanabe K Mizukami Y Muto J. Anti-inflammatory effects of differential molecular weight hyaluronic acids on UVB-induced calprotectin-mediated keratinocyte inflammation. J Dermatol Sci. 2022;107:24-31. 35717315 10.1016/j.jdermsci.2022.06.001

[3] Goldinger SM Stieger P Meier B Micaletto S Contassot E French LE Dummer R. Cytotoxic cutaneous adverse drug reactions during anti-PD-1 therapy. Clin Cancer Res. 2016;22:4023-4029. 26957557 10.1158/1078-0432.CCR-15-2872

[4] Saw S Lee HY Ng QS. Pembrolizumab-induced Stevens-Johnson syndrome in non-melanoma patients. Eur J Cancer. 2017;81:237-239. 28438440 10.1016/j.ejca.2017.03.026

[5] Storandt M Seth R. A case of Stevens-Johnson syndrome/toxic epidermal necrolysis in a patient receiving chemo-immunotherapy with pemetrexed and pembrolizumab. Curr Probl Cancer Case Rep. 2021;3:100048.

[6] Hwang A Iskandar A Dasanu CA. Stevens-Johnson syndrome manifesting late in the course of pembrolizumab therapy. J Oncol Pharm Pract. 2019;25:1520-1522. 30086678 10.1177/1078155218791314

[7] Kumar R Bhandari S. Pembrolizumab induced toxic epidermal necrolysis. Curr Probl Cancer. 2020;44:100478. 31122669 10.1016/j.currproblcancer.2019.05.001

[8] Robinson S Saleh J Curry J Mudaliar K. Pembrolizumab-induced Stevens-Johnson syndrome/toxic epidermal necrolysis in a patient with metastatic cervical squamous cell carcinoma: a case report. Am J Dermatopathol. 2020;42:292-296. 31567395 10.1097/DAD.0000000000001527

[9] Wu JY Kang K Yi J Yang B. Pembrolizumab-induced Stevens-Johnson syndrome in advanced squamous cell carcinoma of the lung: a case report and review of literature. World J Clin Cases. 2022;10:6110-6118. 35949835 10.12998/wjcc.v10.i18.6110 PMC9254208

[10] Khalili B Bahna SL. Pathogenesis and recent therapeutic trends in Stevens-Johnson syndrome and toxic epidermal necrolysis. Ann Allergy Asthma Immunol. 2006;97:272–280; quiz 281-273, 320. 17042130 10.1016/S1081-1206(10)60789-2

[11] Hsu YO Lu KL Fu Y Wang C-W Lu C-W Lin Y-F Chang W-C Yeh K-Y Hung S-I Chung W-H Chen C-B. The roles of immunoregulatory networks in severe drug hypersensitivity. Front Immunol. 2021;12:597761. 33717075 10.3389/fimmu.2021.597761 PMC7953830

[12] Gandhi L Rodríguez-Abreu D Gadgeel S Esteban E Felip E De Angelis F Domine M Clingan P Hochmair MJ Powell SF Cheng SY-S Bischoff HG Peled N Grossi F Jennens RR Reck M Hui R Garon EB Boyer M Rubio-Viqueira B Novello S Kurata T Gray JE Vida J Wei Z Yang J Raftopoulos H Pietanza MC Garassino MC; KEYNOTE-189 Investigators. Pembrolizumab plus chemotherapy in metastatic non-small-cell lung cancer. N Engl J Med. 2018;378:2078-2092. 29658856 10.1056/NEJMoa1801005

[13] Haratake N Tagawa T Hirai F Toyokawa G Miyazaki R Maehara Y. Stevens-Johnson syndrome induced by pembrolizumab in a lung cancer patient. J Thorac Oncol. 2018;13:1798-1799. 29885481 10.1016/j.jtho.2018.05.031

[14] Sassolas B Haddad C Mockenhaupt M Dunant A Liss Y Bork K Haustein UF Vieluf D Roujeau JC Le Louet H. ALDEN, an algorithm for assessment of drug causality in Stevens-Johnson syndrome and toxic epidermal necrolysis: comparison with case-control analysis. Clin Pharmacol Ther. 2010;88:60-68. 20375998 10.1038/clpt.2009.252

[15] Lonjou C Borot N Sekula P Ledger N Thomas L Halevy S Naldi L Bouwes-Bavinck J-N Sidoroff A de Toma C Schumacher M Roujeau J-C Hovnanian A Mockenhaupt M; RegiSCAR study group. A European study of HLA-B in Stevens-Johnson syndrome and toxic epidermal necrolysis related to five high-risk drugs. Pharmacogenet Genomics. 2008;18:99-107. 18192896 10.1097/FPC.0b013e3282f3ef9c

[16] Charlton OA Harris V Phan K Mewton E Jackson C Cooper A. Toxic epidermal necrolysis and Steven-Johnson syndrome: a comprehensive review. Adv Wound Care (New Rochelle). 2020;9:426-439. 32520664 10.1089/wound.2019.0977 PMC7307670

[17] Liotti L Caimmi S Bottau P Bernardini R Cardinale F Saretta F Mori F Crisafulli G Franceschini F Caffarelli C. Clinical features, outcomes and treatment in children with drug induced Stevens-Johnson syndrome and toxic epidermal necrolysis. Acta Biomed. 2019;90:52-60. 10.23750/abm.v90i3-S.8165 PMC6502171 30830062

[18] Nguyen DV Chu HC Nguyen DV Phan MH Craig T Baumgart K van Nunen S. HLA-B*1502 and carbamazepine-induced severe cutaneous adverse drug reactions in Vietnamese. Asia Pac Allergy. 2015;5:68-77. 25938071 10.5415/apallergy.2015.5.2.68 PMC4415182

[19] van Nguyen D Chu HC Vidal C Fulton RB Nguyen NN Quynh Do NT Tran TL Nguyen TN Thu Nguyen HT Chu HH Thanh Thuc HT Minh Le HT van Nunen S Anderson J Fernando SL. Genetic susceptibilities and prediction modeling of carbamazepine and allopurinol-induced severe cutaneous adverse reactions in Vietnamese. Pharmacogenomics. 2021;22:1-12. 33356553 10.2217/pgs-2019-0146

[20] Chantarangsu S Mushiroda T Mahasirimongkol S Kiertiburanakul S Sungkanuparph S Manosuthi W Tantisiriwat W Charoenyingwattana A Sura T Chantratita W Nakamura Y. HLA-B*3505 allele is a strong predictor for nevirapine-induced skin adverse drug reactions in HIV-infected Thai patients. Pharmacogenet Genomics. 2009;19:139-146. 19104471 10.1097/FPC.0b013e32831d0faf

