# Supplementary material for: Pembrolizumab-induced Stevens-Johnson syndrome/Toxic Epidermal Necrolysis in a Vietnamese patient with nonsmall-cell lung cancer
Source: Asia Pac Allergy. 2023 Dec 18;14(2):84–9. doi: 10.5415/apallergy.0000000000000131 (PMC11142754; doi:10.5415/apallergy.0000000000000131)
Supplement: Supplementary file 1 [file pa9-14-84-s001.pdf]

CASE REPORT: PEMBROLIZUMAB-INDUCED STEVENS -JOHNSON SYNDROME/ TOXIC EPIDERMAL NECROLYSIS IN A VIETNAMESE PATIENT WITH NON-SMALL CELL LUNG CANCER

Yen T H Pham, MD,MSc [1]; Mai T Vu, MD, MSc[1,2], Anh Q Nguyen, MD, MSc[1,2], ; Phat N Trinh, MD, MSc [1] ; Mai H Tran, MSc [3]; Hieu C Chu, MD [1,4], Nguyet T M Nguyen, PhD [5]; Chi H V Vu, MD, PhD [6]; Dinh, V Nguyen, MD, PhD[1,2,5,7,*]

[1]Center of Allergy and Clinical Immunology, Vinmec Times City, Vinmec Healthcare System, Hanoi, Vietnam

[2]College of Heath Sciences, VinUniversity, Hanoi, Vietnam

[3]Vingroup Big Data Institute, Hanoi, Vietnam

[4]Center of Allergy and Clinical Immunology, Bach Mai Hospital, Hanoi, Vietnam

[5]Vinmec- VinUni Institute of Immunology, Vinmec Healthcare System, Hanoi, Vietnam

[6]National Ophthalmology Hospital, Hanoi, Vietnam

[7]Department of Medicine, Penn State University, USA

Sup Table 1: Medication timeline

**April**																				**May**																					

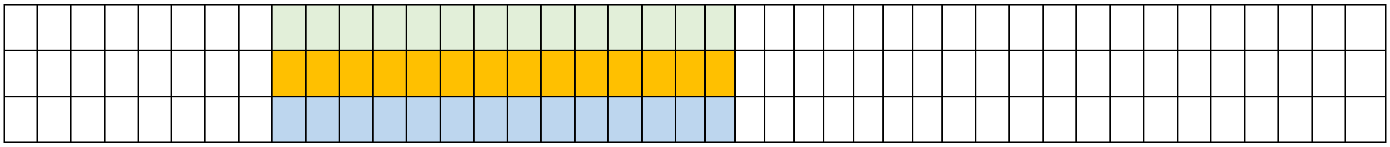

Esomeprazole continued to be used in the hospital without any effect on patient’s clinical symptoms.

	Palitaxel
	Carboplatin
	Pembrolizumab
	Dexamethasone
	Granisetron
	Pregabalin
	Etoricoxib
	Esomeprazole

Sup Table 2: HLA types

HLA class	Genotypes
HLA_A	A*24:02:102:01
	A*02:03:01
HLA_B_	B*35:05:02
	B*38:02:01:01
HLA_C	C*04:01:43
	C*07:02:01:01
HLA_DPA1	DPA1*01:03:01:01
	DPA1*02:01:02:01
HLA_DPB1	DPB1*04:01:01:01
	DPB1*05:01:01:01
HLA_DQA1	DQA1*06:01:01:01
	DQA1*06:01:01:01

HLA_DQB1	DQB1*03:01:01:01
	DQB1*03:01:01:01
HLA_DRB1	DRB1*12:02:01:01
	DRB1*12:02:01:01
